# “Wait and See” Approach to the Emergency Department Cardioversion of Acute Atrial Fibrillation

**DOI:** 10.1155/2011/545023

**Published:** 2011-11-17

**Authors:** Brian Doyle, Mark Reeves

**Affiliations:** Emergency Department, North West Regional Hospital, and Rural Clinical School, University of Tasmania, Burnie TAS 7320, Australia

## Abstract

*Objective*. Acute atrial fibrillation often spontaneously resolves. This study aimed to investigate the outcomes and satisfaction of an evidence-based ED protocol employing a “wait and see” approach. *Methods*. A prospective observational cohort study of adult patients presenting to the Emergency Department with stable acute atrial fibrillation was performed. Patients were excluded if they were considered to be unstable, need hospitalization, or poor candidates for ED procedural sedation. Routine care was provided on the index visit, and suitable candidates were discharged and asked to return to the ED the following day for possible electrical cardioversion. Outcome measures included spontaneous reversion to sinus rhythm, success of cardioversion, length of stay, adverse event and return visits for AF within 30 days, and patient satisfaction. *Results*. Thirty five patient encounters were analysed over a 21-month period. Twenty two of the 35 patients (63%) had spontaneous resolution of atrial fibrillation upon presentation for potential cardioversion. All of the remaining patients underwent successful cardioversion to normal sinus rhythm without significant adverse events recorded. No patients required hospitalization. Three patients (9%) returned to the ED within 30 days for recurrence of atrial fibrillation. All patients were reported to be “very satisfied” with this approach. *Conclusion*. A “wait and see” approach to the ED electrical cardioversion of atrial fibrillation showed that almost two-thirds of patients had spontaneous resolution without requiring cardioversion or observation in the ED or hospital. All patients were successfully reverted to normal sinus rhythm and had a high degree of satisfaction.

## 1. Introduction

Atrial fibrillation (AF) is the most commonly encountered arrhythmia in the Emergency Department (ED). There are quite wide differences in the ED management of acute AF [1–3]. Treatment primarily focuses on rate or rhythm control along with anticoagulation if necessary. Traditionally, many patients were hospitalized when found to be in acute AF. More recent studies have demonstrated the safe cardioversion of selected patients with acute AF in the Emergency Department without requiring hospitalization [4–8]. Others have shown the suitability for these patients to be treated successfully in an observation unit [9, 10].

Well-regarded international guidelines allow for the cardioversion of acute AF in selected patients within a 48-hour window of opportunity before anticoagulation must be instituted [11]. However, there is debate as to the appropriate strategy of rate or rhythm control for acute atrial fibrillation [12, 13]. Nevertheless, cardioversion of acute AF has become an accepted practice in many Emergency Departments [2].

Numerous studies quite clearly demonstrate the superiority of electrical cardioversion over pharmacologic cardioversion for obtaining sinus rhythm [1, 2, 4–7, 11]. Disadvantages of pharmacological attempts include pro-arrhythmic side effects of medications, longer time in the ED, and lower overall success rate. The primary disadvantage of DC cardioversion relates to the procedural sedation. 

The likelihood of spontaneous conversion of acute AF to sinus rhythm is reported to be as high as 70% [14]. Although active cardioversion of patients is generally considered to be safe, one could reasonably question the strategy of immediate treatment for a condition that is likely to resolve spontaneously. In this age of access block and limited resources, it is important to create protocols that maximize existing resources and ensure patient safety. 

An evidence-based protocol was created at a single institution that allowed for the potential spontaneous resolution of AF prior to active attempts at cardioversion. To the best of our knowledge, this “wait and see” approach has not been previously studied. The protocol allowed for the early discharge of patients without prolonged observation or admission to hospital. The study assessed the outcomes and satisfaction of this protocol.

## 2. Methods

A prospective observational cohort study was undertaken to investigate the outcomes and satisfaction of an evidence-based protocol (Table 1) for the treatment of acute atrial fibrillation. Formal approval was obtained from the Tasmanian Human Research Ethics Network.

The North West Regional Hospital in Tasmania, Australia is formally accredited for training in Emergency Medicine and has an annual census of 25,000 visits. This study was undertaken for a 21-month period from December 2008 to August 2010. 

Adult patients were approached for inclusion at the discretion of the treating doctor if they were considered to have stable acute AF and suitable for a rhythm control strategy. Patients were excluded if they were considered to be unstable, have severe symptoms, require hospitalization, pregnant, or poor candidates for ED procedural sedation. It was required that the protocol and potential cardioversion be completed within the safe 48-hour window of opportunity for this procedure. 

The protocol called for participants to be given usual treatment for rate control and anticoagulation at the discretion of the initial treating doctor. The lead study investigator (BD) was contacted upon patient enrolment and a standard data form was completed. The participants were discharged and asked to return the following day to the ED for potential electrical cardioversion. Institutional guidelines for procedural sedation were followed. All procedures were conducted by Emergency Medicine specialists (Fellows of the Australasian College of Emergency Medicine). The agent(s) for procedural sedation and the energy level of each shock were at the discretion of the treating doctor. 

Data were initially collected regarding patient demographics and medications given in the Emergency Department. Data collected on the second visit included the agents of procedural sedation given, number of shocks administered, and joules per shock. ED length of stay was calculated. 

Study outcomes included the proportion of patients with spontaneous resolution of AF upon presentation for potential cardioversion, the success of DC cardioversion, the overall return to sinus rhythm, and the number of patients returning to the ED within 30 days with a diagnosis of atrial fibrillation. Patient satisfaction was determined on a five-point modified Likert scale conducted by telephone interview at 7 days by the lead investigator. Prespecified adverse events included oxygen desaturation less than 90% during procedural sedation, aspiration, utilization of bag valve mask, endotracheal intubation, apnoea greater than 30 seconds, and acute stroke. Other complications not prospectively defined were also sought. 

Data were entered and analysed in to Microsoft Office Excel 2003 for Windows (Microsoft Corporation, Seattle, USA). Standard descriptive statistics were derived; ED lengths of stay (EDLOS) were expressed as median and interquartile range (IQR). Comparisons of EDLOS were made using Wilcoxon signed-rank tests using Stata 10.0 (StataCorp, Tex, USA).

## 3. Results

During the study period, 256 patients were discharged or admitted from the Emergency Department with a final diagnosis of atrial fibrillation of any duration. Most of these patients did not have acute AF and/or met other exclusion criteria. Thirty-four adult patients consented to inclusion but one patient was withdrawn for meeting exclusion criteria. Two patients were enrolled twice during the study period. There were thirty-five evaluable datasets. The overall results are summarized on Table 2. 

Twenty-two (63%) patients had spontaneous resolution of atrial fibrillation discovered upon representation on day 2 and thus did not require electrical cardioversion. All remaining patients underwent successful DC cardioversion. All patients were discharged from the ED on day 2 in sinus rhythm. Six patients returned to the ED within 30 days, of whom 3 (9%) were found to be in atrial fibrillation. The median total ED length of stay combined for both visits was 177 (IQR 137–260) minutes. For those that spontaneously reverted their median total time was 150 (IQR 105–193) minutes and for those that required cardioversion 263 (IQR 221–304) minutes (*P* < 0.001). No patients required hospitalization. All patients (100%) were reported to be “very satisfied” upon telephone followup at 7 days after treatment

All procedural sedations were performed using propofol with an average total dose of 1 mg/kg. Median number of shocks was 1 and ranged from 1–4.

There were no prespecified adverse events reported. Unstructured adverse events included one report of “asymptomatic hypotension” during procedural sedation. One patient reported a “sore chest” during telephone interview at day 7. There were no cases of stroke.

## 4. Discussion

The treatment strategy of acute atrial fibrillation is not clearly defined. Several randomized trials comparing rate to rhythm control have not shown an advantage to rhythm control [15–17]. However, there are several limitations to these studies. Many enrolled older patients with more persistent atrial fibrillation. This may not be externally valid to a younger patient with short onset of atrial fibrillation that may present to the Emergency Department. 

There is a common saying “atrial fibrillation begets atrial fibrillation” [11]. This implies that there is atrial remodelling in response to AF that perpetuates the arrhythmia. Well-regarded published guidelines conclude that there is a window of opportunity to maintain sinus rhythm that should not be overlooked early in the course of management of AF. Rate control may be a reasonable option in older patients with persistent AF, but for the younger patient, especially those with paroxysmal lone AF, rhythm control may be the better initial option. These guidelines state that cardioversion can be safely performed without preceding anticoagulation in suitable patients if AF is of less than 48-hour duration [11]. 

The rate of spontaneous conversion of acute atrial fibrillation is high. A prospective study of spontaneous conversion rates of acute AF (<72 hours duration) to sinus rhythm showed almost a 70% conversion rate [14]. Patients with symptoms of less than 24 hours duration were the best predictor of spontaneous conversion. Many other studies looking at pharmacologic agents versus placebo for cardioversion give us a clue as to the rate of spontaneous conversion of atrial fibrillation [18–21]. A randomized, placebo controlled trial of amiodarone demonstrated a 64% conversion rate within 24 hours in those that received placebo [18]. A placebo controlled study of propafenone versus digoxin plus quinidine showed a 76% conversion rate in the placebo arm [19]. One might conclude that “placebo” is quite effective and safe for the treatment of acute atrial fibrillation. 

If the spontaneous conversion rate of acute atrial fibrillation is quite high, then one should seriously question a strategy that involves early active treatment. This may expose the patient to unnecessary procedures and medications, potential complications, and unnecessarily utilize hospital resources. Our study employed a “wait and see” approach to allow for the spontaneous reversion of AF while the patient was discharged home. Almost two-thirds of the patients did not require active treatment with electrical cardioversion. Total ED length of stay was short compared to other published reports as described above, and all patients were eventually discharged in sinus rhythm.

There are several limitations to this study. The number of patients enrolled was small. This does not allow for detection of less common complications. The success rate of 100% to sinus rhythm is contrary to other published reports showing rates closer to 90% for electrical cardioversion. The median age of our study population was 57 compared to the average age of patients with AF reported to be around 75 years of age. This was not a trial of all patients with acute AF, and clearly a younger and likely healthier population was selected. This study was performed at a single site with motivated investigators and external validity must be considered. All procedural sedation was undertaken by Emergency Medicine specialists and other outcomes might be observed utilizing other groups. In general, ED length of stay tends to be shorter in regional hospitals. A reported high degree of patient satisfaction may represent some bias as this telephone interview was conducted by the lead investigator who may have also been directly involved in their patient care. 

The protocol did not specify which medications should be given for rate control and/or anticoagulation. Three patients received amiodarone and one patient sotolol which may account for higher conversion rates.

There is no patient charge for public ED care in Australia. Therefore external validity must be considered when addressing satisfaction and compliance with this protocol in other settings. 

One might reasonably question a protocol requiring patients to return to the ED for a scheduled elective cardioversion. However, in this study admission to hospital or observation unit was avoided. In addition, owing to the natural history of acute AF, almost two-thirds of patients did not require active cardioversion. Other institutions may have other resources available to allow for a different approach. Therefore, this study may not be applicable or feasible in other departments. But it would be difficult to imagine a strategy at most hospitals that would use less resources, decrease admissions, decrease ED length of stay, minimize procedures, all the while resulting in reversion to normal sinus rhythm.

## 5. Summary

This study of a “wait and see” protocol for the ED cardioversion of acute atrial fibrillation demonstrated almost two-thirds of patients had spontaneous resolution of AF prior to active attempts at cardioversion. Total ED length of stay was short, and all patients attained sinus rhythm. Patients did not require admission to hospital or an observation ward. Adverse events were minimal. A small percentage (9%) returned within 30 days with a recurrence of AF. This strategy met with a high degree of patient satisfaction. 

## Figures and Tables

**Table 1:** Emergency Department protocol for cardioversion of atrial fibrillation.

(i) Ensure patient has stable acute atrial fibrillation	
(ii) Exclusion criteria: unstable or severe symptoms, patient requires hospitalization, >48-hour duration of AF by next day cardioversion, poor candidate for ED procedural sedation, age <18, or pregnancy	
(iii) Patients may get rate control and other medications at the discretion of treating doctor	
(iv) Discharge patient to return to ED the following day at 08:00 for DC cardioversion. Advise patient to fast after midnight.	
(v) Obtain ECG on day 2	
(vi) If patient still in AF, confirm inclusion & exclusion criteria and proceed with DC cardioversion	

**Table 2:** Demographics & ED Length of Stay.

	Spontaneous reversion (*n* = 22)	Not spontaneously reverted (*n* = 13)
Gender (M : F)	14 : 8	9 : 4
Median age (IQR) in years	55 (44–66)	62 (51–63)
EDLOS day 1 in minutes (IQR)	124 (84–141)	151 (111–187)
EDLOS day 2 in minutes (IQR)	24 (15–45)	96 (70–130)*
EDLOS total in minutes (IQR)	150 (105–193)	263 (221–304)**

*P < 0.000, **P < 0.001.
